# Magnetocardiography for noninvasive surveillance of rejection and cardiac allograft vasculopathy in heart transplant recipients^☆^

**DOI:** 10.1016/j.ahjo.2025.100704

**Published:** 2025-12-23

**Authors:** Christian Eskander, Matthew Peters, Dipankar Gupta, Katelyn A. Bruno, Juan R. Vilaro

**Affiliations:** a Department of Internal Medicine, University of Florida, Gainesville, Florida, USA; b Congenital Heart Center, University of Florida, Gainesville, Florida, USA; c Department of Pediatrics, University of Florida, Gainesville, Florida, USA; d Division of Cardiovascular Medicine, Department of Medicine, Gainesville, Florida, USA

## Abstract

Heart transplantation is the definitive treatment for end-stage heart failure, yet long-term graft survival is hindered by two major complications: acute/chronic rejection (cellular or antibody mediated) and cardiac allograft vasculopathy (CAV). Standard surveillance is performed to screen for these issues and predominantly consists of cardiac catheterization with hemodynamics, endomyocardial biopsy and invasive coronary angiography, with intravascular ultrasound (IVUS) and coronary flow reserve. The use of IVUS increases the sensitivity for CAV detection. Overall, these procedures have associated morbidities, need anesthesia and have associated patient discomfort and substantial cost. Magnetocardiography (MCG), a noninvasive modality that measures cardiac magnetic fields, has emerged as a potential tool for early detection of complications in post-transplant patients. Unlike electrocardiography, MCG provides spatially resolved data on depolarization and repolarization, independent of body habitus or tissue conductivity. Early studies suggest that MCG can identify electrophysiologic abnormalities associated with both acute rejection and CAV, in some cases preceding histologic or angiographic confirmation. Rejection is reflected by alterations in magnetic dipole strength and repolarization heterogeneity, while CAV correlates with repolarization dispersion indices such as QTc heterogeneity and Magnetic Dispersion Velocity. Despite promising pilot data, MCG remains underutilized, largely due to small study sizes, lack of standardized interpretation, and limited availability of equipment. This review synthesizes the existing evidence, highlights potential advantages and limitations, and outlines future directions for integrating MCG into standard post-transplant surveillance protocols.

## 1 Introduction

Heart transplantation remains the gold standard for patients with advanced, refractory heart failure. Improvements in surgical techniques and immunosuppressive therapy have extended median survival, yet long-term graft success continues to be challenged by acute/chronic rejection (cellular or antibody mediated) and cardiac allograft vasculopathy (CAV). Acute rejection, particularly within the first year, carries a significant morbidity and mortality if not promptly identified and treated. CAV, on the other hand, usually develops progressively over time, manifesting as diffuse concentric intimal hyperplasia and representing the leading cause of late graft failure.

Current surveillance relies heavily on invasive procedures. Endomyocardial biopsy (EMB) is considered the gold standard for diagnosis of rejection, but it is associated with procedural risks, including myocardial perforation, tricuspid valve regurgitation secondary to valve leaflet or support structure injury, and arrhythmias. Moreover, its diagnostic yield is constrained by sampling error, especially with repeated biopsies as myocardial fibrosis increases and often times the patchy nature of rejection. For the diagnosis of CAV, coronary angiography and intravascular ultrasound (IVUS) remain the most commonly performed studies. These procedures, while informative, are invasive, expensive, and burdensome for patients who may require serial surveillance over years of follow-up.

Therefore, there is an urgent need for a safe, reliable, and reproducible noninvasive monitoring tool to aid in the early diagnosis of these complications. Magnetocardiography (MCG) has re-emerged as a potential modality capable of filling this gap. By measuring the magnetic fields generated by myocardial depolarization and repolarization, MCG provides a three-dimensional and highly sensitive assessment of cardiac electrophysiology. Modern advances in sensor technology allow these measurements to be obtained without shielding or contrast administration. In principle, MCG offers a unique opportunity: to identify subtle physiologic disturbances associated with rejection or CAV before irreversible damage occurs. This review critically evaluates the current evidence supporting MCG in heart transplantation, with a focus on its application in rejection and CAV detection.

### 1.1 Magnetocardiography: principles and clinical features

**Image 1:**
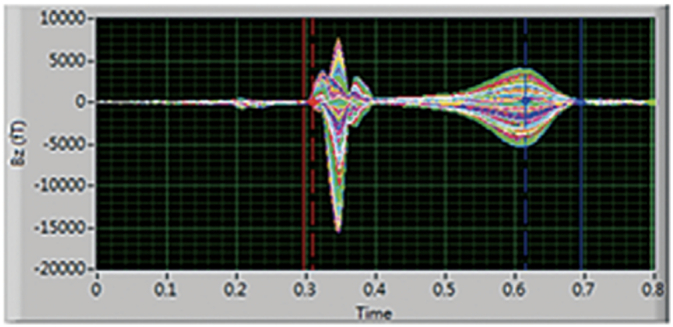
- MCG Butterfly Plot [1].

**Image 2:**
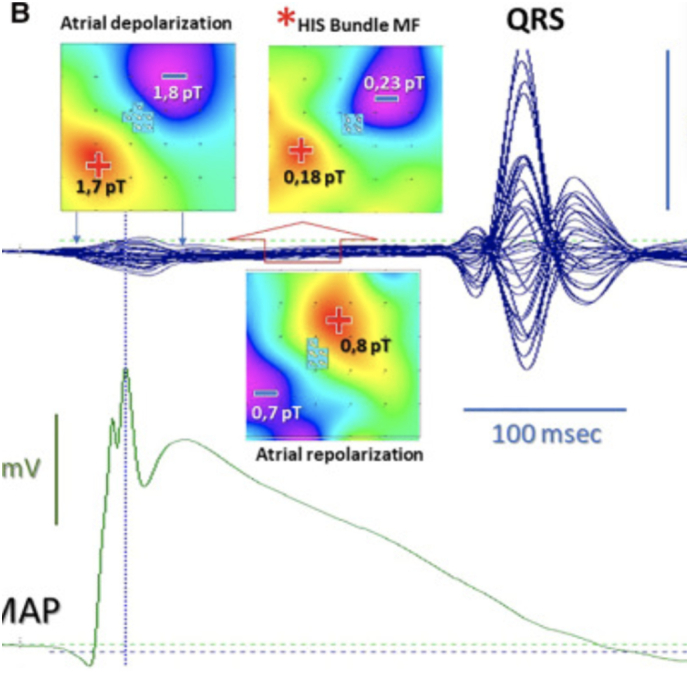
- Example of an MFM [2].

**Image 3:**
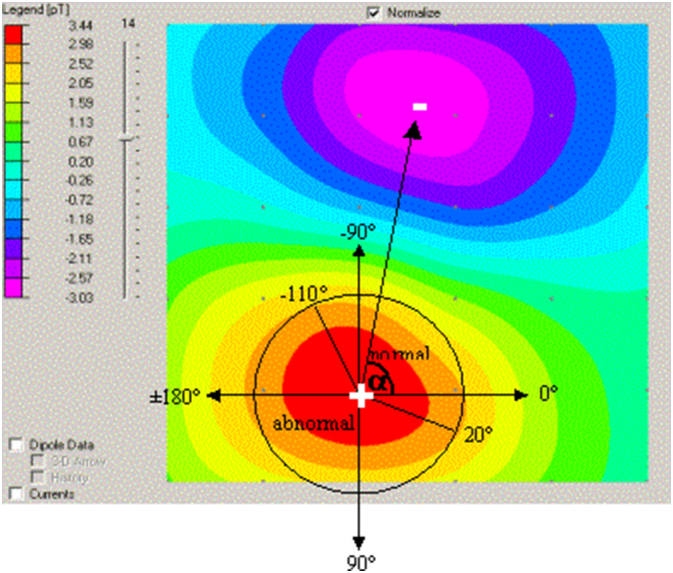
- Examples of spatial mapping results [3].

**Image 4:**
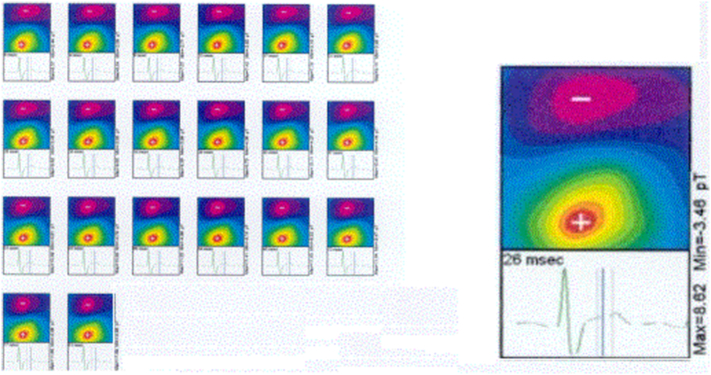
- Examples of spatial mapping results [3].

MCG captures the magnetic signatures generated by ionic currents within the myocardium. These signals, measured by arrays of superconducting or optically pumped magnetometers, are visualized as butterfly plots (Image 1 and Image 2) and converted into magnetic field maps (Image 3 and Image 4). These maps depict the distribution and polarity of electrical activity, allowing clinicians to assess repolarization and depolarization dynamics in a manner not possible with the traditional surface electrocardiogram (ECG). Examples of the MCG device are seen in Image 7, Image 8.

**Image 5:**
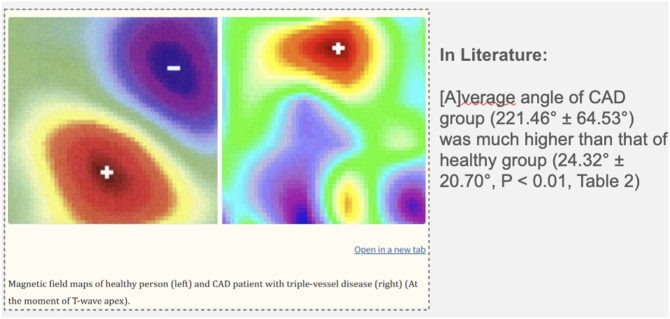
- RT Angle in healthy group (left) vs CAD group (right) [7].

Key electrophysiologic markers derived from MCG include magnetic field strength and velocity, Magnetic Dispersion Velocity, repolarization coordination, and the RT angle (Image 5). Deviations in these parameters often manifest as multipolarity of the magnetic field, abnormal T-wave heterogeneity, or ST-segment abnormalities. In healthy hearts, repolarization is spatially homogeneous, but in disease states, these orderly patterns are disrupted. Importantly, such changes may be reversible, as demonstrated by the restoration of dipole alignment after revascularization.

While MCG has shown diagnostic promise in ischemic heart disease, coronary microvascular dysfunction, and cardiomyopathy [6,7], its most compelling potential may lie in the field of cardiac transplantation, with serial evaluations and comparing evolution over time, allowing identification of subtle changes which are unable to be picked up with the electrocardiogram and other routine non-invasive testing.

### 1.2 MCG in rejection

Rejection is immune-mediated and is typically classified as either cellular or antibody-mediated. Its surveillance requires frequent EMBs during the first post-transplant year, a practice that is both burdensome and imperfect. Biopsies may miss focal rejection, and their diagnostic yield diminishes over time.

MCG offers an alternative by detecting physiologic changes in myocardial conduction that occur during rejection. In early work from Berlin, Schmitz et al. reported that MCG abnormalities correlated with histologic rejection, achieving sensitivity of 91 % and specificity of 93 % [4]. Fenici and colleagues similarly observed that serial MCGs could detect rejection earlier than EMB in certain patients, particularly when immunosuppression was subtherapeutic [2]. Achenbach later confirmed these findings with 83 % sensitivity and 84 % specificity in a small cohort [8], while Fernando presented a case report in which MCG findings aligned with both EMB and coronary angiography [3].

Tavarozzi and colleagues expanded on this literature, emphasizing that MCG could identify rejection-related electrophysiologic changes but cautioning that standardization of diagnostic criteria was essential before routine clinical adoption [5]. The recurring theme across all these studies suggests that rejection manifests as increased repolarization heterogeneity, abnormal dispersion velocities, and altered magnetic dipole configurations on MCG evaluation.

Although these findings are compelling, they must be interpreted with caution, especially in the context of small sample sizes and predominantly single-center experiences. Nonetheless, MCG may be most valuable in the surveillance of clinically stable patients, where a normal scan could reduce the need for scheduled surveillance biopsies.

### 1.3 MCG in cardiac allograft vasculopathy

CAV represents the leading cause of late mortality and graft loss after transplantation. Its diffuse and concentric nature makes it difficult to detect early with conventional angiography, which visualizes luminal narrowing rather than intimal thickening. IVUS offers higher sensitivity; however, it is still limited to proximal larger coronary arteries and falls short of assessing microvascular disease. Additionally, these techniques allow higher sensitivity but at the expense of greater invasiveness and complexity. Similarly, coronary flow reserve assesses the increase in coronary flow with administration of adenosine when compared to baseline, allowing a functional assessment of coronary vasodilation.

**Image 6:**
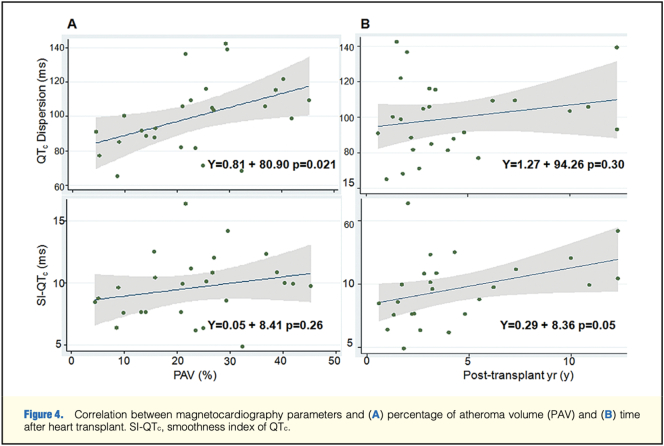
- Correlation b/w MCG heterogeneity and percentage of atheroma volume and time after heart transplant [1].

MCG has shown potential in this domain by quantifying repolarization heterogeneity. Wu et al. demonstrated that QTc dispersion and the smoothness index derived from MCG correlated strongly with plaque burden measured by IVUS in 26 transplant recipients [1] (Image 6). Li et al. extended these findings to coronary artery disease, reporting favorable diagnostic accuracy [7]. Collectively, these studies suggest that MCG can detect microvascular dysfunction before overt angiographic abnormalities become apparent.

**Image 7:**
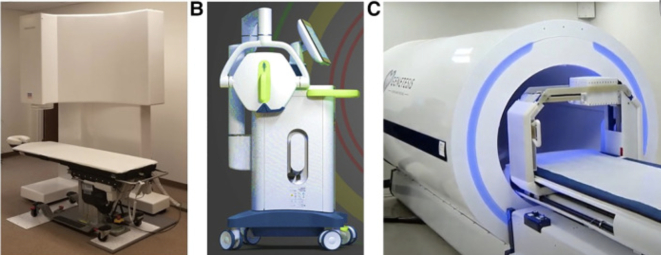
MCG device [2].

**Image 8:**
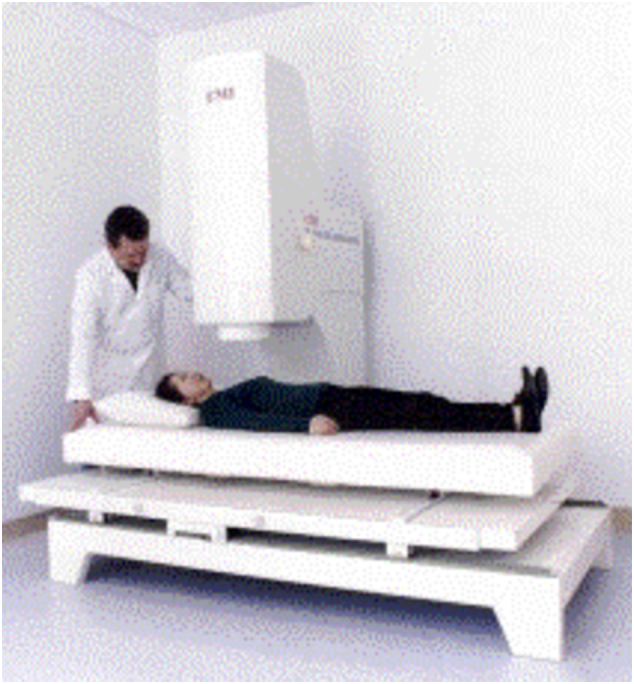
MCG Device with a patient [3].

Clinically, this could be an important breakthrough in the early diagnosis of CAV. Once validated, MCG could serve as a noninvasive screening tool for CAV, reducing reliance on serial angiography in stable patients and enabling earlier therapeutic interventions. However, as with rejection, the evidence for CAV detection remains preliminary, limited by small sample sizes and heterogeneity in methodology.

### 1.4 MCG in pediatric patients

Pediatric heart transplant patients are a unique group and the current median survival for infant transplants is about 20 years. In contrast to adult patients, pediatric patients have a mix of congenital heart disease and cardiomyopathy as the reason for transplantation. With increasing age, the proportion of cardiomyopathy as a reason for transplantation increases, while in smaller children, congenital heart disease is the predominant reason for transplantation. Pediatric patients are commonly plagued by difficult access, need for sedation and potential complications such as perforation and valve injury may be more likely. Furthermore, many patients may have chronic occlusion in their blood vessels, limiting the ability to even obtain a coronary assessment. MCG in pediatric patients has been utilized for monitoring the electrical activity of the fetal heart and diagnosing heart rhythm and conduction abnormalities. In fact, there is currently an open NIH-funded trial at the Children's Hospital of Wisconsin, where investigators are using mobile fetal magnetocardiography to define fetal heart conduction. Additionally, even in younger children, the MCG may be of significant benefit as the scan times are quick, eliminating or minimizing the need for sedation. Overall, the cumulative burden of the regular surveillance cardiac catheterizations and endomyocardial biopsies throughout a child's life span is quite significant. The additional burden of anxiety and the emotional distress caused by the upcoming procedure and the associated risks and complications seem overwhelming for the patients and their families. MCG may serve as a critical tool that will allow to bridge this gap by promoting non-invasive diagnosis.

### 1.5 Advantages, limitations, and future directions

The appeal of MCG lies in its safety, repeatability, and sensitivity to subtle physiologic changes. Unlike EMB, it is noninvasive and free from procedural risks. Unlike angiography, it requires no contrast or radiation. Its spatial resolution exceeds that of ECG, and it is unaffected by thoracic conductivity or body habitus. These features make it particularly suited for the transplant population, which requires frequent, long-term longitudinal monitoring.

Despite the promising technology, significant barriers remain. The current literature is composed almost entirely of small, single-center studies with variable methodology [1–6,13]. The interpretation of MCG data lacks standardization, and diagnostic thresholds, such as SI-QTc or MDV cutoffs, are inconsistently applied. Moreover, access to MCG equipment is limited; however, advances in portable, unshielded systems may significantly improve feasibility.

The diagnostic role of MCG continues to expand in realms beyond the diagnosis of rejection or detection of CAV. Its use is being studied as a tool in the diagnosis of myocarditis and other inflammatory cardiomyopathies, in the detection of ischemic heart disease, or to predict the need for implantable cardioverter defibrillators (ICD) based on arrhythmogenic features noted on MCG to name a few current areas of investigation [[9], [10], [11]]. Just as there are multiple possible utilizations of MCG in cardiovascular care, there are multiple MCG devices currently commercially available as well as being used in active clinical research. Future areas of MCG development and research include the increased use of unshielded and portable systems, the development of wearable devices for ambulatory patients, as well as the integration of artificial intelligence in signal analysis.

Future directions include integrating MCG with other non-invasive biomarkers, such as donor-derived cell-free DNA, cardiac MRI, and echocardiographic strain imaging. Artificial intelligence holds promise for automated analysis of MCG waveforms, potentially improving reproducibility and diagnostic accuracy. Importantly, cost-effectiveness studies are needed to determine whether MCG can also reduce healthcare utilization by lowering the frequency of invasive surveillance procedures.

## 2 Conclusion

Magnetocardiography represents a promising frontier in the surveillance of heart transplant recipients. Early data suggest that it can identify rejection and CAV with good diagnostic accuracy, often before abnormalities are apparent on biopsy or angiography. Its ability to detect subtle physiologic disturbances, combined with its safety and repeatability, makes it an attractive adjunct to current surveillance paradigms. However, the field remains in its infancy. Large, multicenter studies are necessary to validate diagnostic thresholds, standardize interpretation, and establish the cost-effectiveness of these approaches. Once these hurdles are overcome, MCG could fundamentally reshape post-transplant monitoring, sparing patients the risks and burdens of invasive testing while potentially improving long-term outcomes.

## CRediT authorship contribution statement

**Christian Eskander:** Conceptualization, Writing – original draft, Writing – review & editing. **Matthew Peters:** Writing – original draft, Writing – review & editing. **Dipankar Gupta:** Writing – original draft, Writing – review & editing. **Katelyn A. Bruno:** Conceptualization, Methodology, Project administration, Supervision, Writing – original draft, Writing – review & editing. **Juan R. Vilaro:** Conceptualization, Project administration, Supervision, Writing – original draft, Writing – review & editing.

## Ethics Statement

None.

## Funding

None.

## Declaration of competing interest

None.
